# Home-based treatment for patients with hematological cancer in Denmark–A national overview

**DOI:** 10.1007/s00520-025-10048-0

**Published:** 2025-10-30

**Authors:** Kristina Holmegaard Nørskov, Hanne Bødtcher, Tine Rosenberg, Camilla Thim Damgaard, Iben Husted Nielsen, Helle Enggaard, Susanne Oksbjerg Dalton

**Affiliations:** 1 Department of Haematology, Zealand University Hospital, Roskilde, Denmark; 2 Department of Regional Health Research, University of Southern Denmark, Odense, Denmark; 3 Danish Cancer Institute, Danish Cancer Society, Copenhagen, Denmark; 4 Department of Hematology, Odense University Hospital, Odense, Denmark; 5 Department of Hematology, Aarhus University Hospital, Aarhus, Denmark; 6 Department of Hematology, Copenhagen University Hospital, Rigshospitalet, Copenhagen, Denmark; 7 Clinical Nursing Research Unit & Clinical Cancer Research Center, Aalborg University Hospital, Aalborg, Denmark; 8 Department of Clinical Oncology and Palliative Care, Zealand University Hospital, Næstved, Denmark; 9 Institute of Clinical Medicine, University of Copenhagen, Copenhagen, Denmark

**Keywords:** Home-based, Hematology, Home, Treatment

## Abstract

**Purpose:**

To investigate the extent and practice of home-based treatment for patients with hematologic malignancies in Denmark.

**Methods:**

This nationwide exploratory study was conducted at all Danish hematologic departments (*n* = 9). Each site received an online questionnaire for the collection of data on the type of therapy administered as home-based treatment, the specific treatments with details on target populations, patient criteria, implementation status, and practice for clinical management.

**Results:**

Six types of therapy were offered as home-based treatments across the Danish hematologic departments: chemotherapy, immunoglobulin, immunotherapy, hydration therapy, antibiotics, and parenteral nutrition. In total, 17 treatments were offered, with the majority being provided to patients with multiple myeloma. Immunoglobulin and antibiotics were most often provided at home. Variations were identified regarding patient criteria for receiving home-based treatment, including personal resources and social networks, use of intravenous accesses and infusion pumps, and timing of initiation of home-based treatment.

**Conclusion:**

There is variation in the implementation of home-based treatment for patients with hematologic malignancies in Denmark, including different criteria and practices. To fully realize the benefits of home-based treatment, it is imperative to address access disparities, standardize practices, and enhance support for patients and caregivers. Continued research, collaboration, and policy development are essential to ensure that all patients have equitable access to high-quality home-based treatment options.

## Introduction

Advancements in treatment modalities, including targeted therapies and immunotherapies, have contributed to improved survival rates among patients with hematologic malignancies [1, 2]. Though these therapeutic innovations have revolutionized the landscape of hematologic oncology, patients undergoing treatment are still at increased risk of life-threatening complications, a significant symptom burden, decreased quality of life (QoL), and of encountering an unpredictable long-term clinical course [3–6]. Moreover, the positive impact on survival rates has contributed to the increasing demand for hematologic services, as more patients require ongoing treatment and long-term care [7].

Currently, most treatments are administered in inpatient or outpatient settings [8]. However, recent advancements in treatment modalities have introduced the concept of home-based treatment, making it possible to provide treatment to patients in their own homes [9–12]. Still, great variation exists in the definition of home-based treatment. In this study, home-based treatment includes outpatient medical and therapeutic procedures carried out in the patient’s own home rather than in the hospital. This differs from the concept of Hospital at Home (HaH), which represents home-based hospital care providing acute, hospital-level care equivalent to conventional inpatient hospitalization [13]. Both HaH and home-based treatment are considered valuable for patients with cancer and the healthcare system [13–15]. Similarly, home-based treatment has gained significant attention in recent years due to its potential to improve patient satisfaction, reduce healthcare costs, and enhance overall QoL [16, 17]. The concept of home-based treatment presents several potential benefits, including enhanced patient-centeredness, psychological well-being, reduced inpatient days and outpatient visits, and support for everyday life activities [10, 11, 18, 19]. Therefore, during the last decade, there has been a significant increase in demand and volume of home-based treatment, which may be highly relevant in a healthcare system with an increasing scarcity of resources. Previous studies have investigated home-based chemotherapy in patients with hematologic malignancies in Denmark and found convincing feasibility, safety, and acceptability [9, 12, 19–22]. These findings align with results of an international review on home-based chemotherapy, which substantiates the beneficial effect of treatment provided in patients’ homes as a promising, safe, and patient-centered alternative to hospital and outpatient care [10]. However, to date, there are no comprehensive overviews of access to home-based treatment interventions for patients with hematologic malignant diseases [10], making it difficult to identify underserved patients and barriers to home-based treatment.

In Denmark, the basic principle of the welfare system is that most healthcare services are tax-funded and provided free of charge, ensuring all residents have equal access and rights to social security and healthcare benefits The treatment for hematologic malignancies is centralized at five university hospitals (UH), which typically handle highly specialized and complex care, and four regional hospitals (RH), which encompass fewer complex conditions still requiring specialized knowledge and equipment. This system helps balance accessibility and the concentration of expertise, ensuring high-quality care across the country. The national guidelines recommend which treatments patients receive based on, e.g., diagnosis and disease stage. However, when it comes to the implementation of home-based treatment programs, no guidelines or cross-national collaborations exist. To further improve the development of home-based treatment programs both nationally and internationally, investigations concerning home-based treatment, specific delivery models, and best practices for clinical and administrative management are needed. Therefore, the present national study was conducted to investigate the extent and practice of home-based treatment for patients with hematologic malignancies in Denmark.

## Methods

This Danish nationwide exploratory study was conducted in accordance with the statement guidelines for reporting observational studies (STROBE) [23]. The study was conducted by the Danish Cancer Institute in collaboration with the Danish Multidisciplinary Hematological Research Network (NIHR-DK).

### Settings and recruitment

In Denmark, there are hematological departments at five larger UH; Aarhus University Hospital (UH1), Aalborg University Hospital (UH2), Copenhagen University Hospital (UH3), Odense University Hospital (UH4), and Zealand University Hospital (UH5) and combined hematological and oncological departments at four RH: Gødstrup Hospital (RH1), Esbjerg Hospital (RH2), Vejle Hospital (RH3), and Hospital of Southern Jutland (RH4) (Table 1). All hematological departments received an email with information about the study and a direct link to an online questionnaire in a RedCap database.

**Table 1:** Hospital demographics and statistics

Hospital	Total inpatient days in 2023 *n* (%)	Total outpatient visits in 2023 *n* (%)
UH1	15,634 (34.8)	33,989 (23.6)
UH2	9457 (21.0)	27,716 (19.2)
UH3	14,453 (32.1)	53,528 (37.1)
UH4	11,829 (26.3)	29,000 (20.1)
UH5	9924 (22.1)	52,000 (36.0)
RH1	3782 (8.4)	12,813 (8.8)
RH2	2922 (6.5)	12,265 (8.5)
RH3	2541 (6.6)	14,348 (9.9)
RH4	0	7458 (5.2)

*UH* University Hospital, *RH* Regional Hospital, *UH2* Aarhus University Hospital, *UH2* Aalborg University Hospital, *UH3* Copenhagen University Hospital, *UH4* Odense University Hospital, *UH5* Zealand University Hospital, *RH1* Gødstrup Hospital, *RH2* Esbjerg Hospital, *RH3* Vejle Hospital, *RH4* Hospital of Southern Jutland

### Data collection and analysis

Data on types of therapies, names, and details of the specific treatments provided as home-based treatment were collected. These included diagnosis (acute myeloid leukemia (AML), acute lymphatic leukemia (ALL), chronic lymphatic leukemia (CLL), chronic myeloid leukemia (CML), multiple myeloma (MM), non-Hodgkin lymphoma (non-HL), Hodgkin lymphoma (HL), and myelodysplastic syndrome (MDS)), patient criteria, year and status of implementation, information on treatment delivery, dispensation, and administration to the patient while being home. Data also comprised total administration days per treatment cycle, a total number of inpatient days or outpatient visits reduced per treatment cycle, and an estimate of the total number of patients receiving the specific treatments as home-based treatment during 2023. Moreover, data on the reasons for patients to opt out or not be qualified for home-based treatment were collected, as well as the timing for the earliest initiation of treatment at home. Finally, the total number of inpatient days and outpatient visits at the hematologic department at each hospital in 2023 was collected.

Basic descriptive statistics were calculated for the clinical characteristics of home-based treatments, and the type of therapy/treatment provided at home across diagnosis and hospitals. The analyses were performed using the software program SPSS [24].

### Ethics

The Danish Data Protection Agency (p-2024–16026) approved the study. All participating hospitals were provided with oral and written study information and informed that participation was voluntary.

## Results

All nine hematologic departments participated and completed the data collection. The total number of inpatient days and outpatient visits for each hospital in 2023 is clarified in Table 1.

**Fig. 1:**
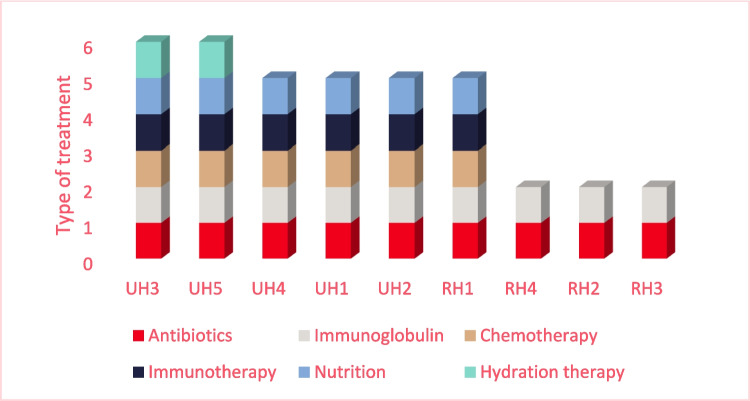
Types of home-based treatment across hospitals. UH; University Hospital, RH; Regional Hospital, UH3; Copenhagen University Hospital, UH5; Zealand University Hospital, UH4; Odense University Hospital, UH1; Aarhus University Hospital, UH2; Aalborg University Hospital, RH1; Gødstrup Hospital, RH2; Esbjerg Hospital, RH3; Vejle Hospital, RH4; Hospital of Southern Jutland

**Fig. 2:**
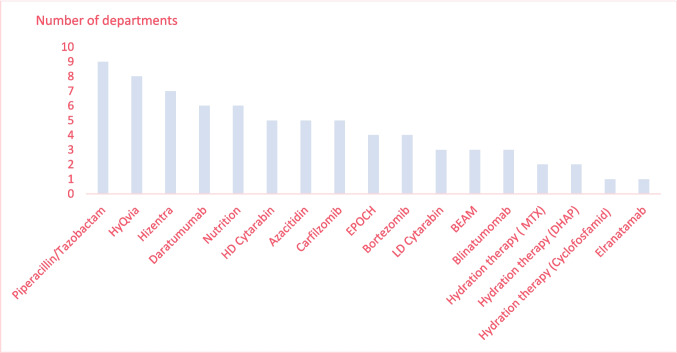
The number of hospitals that provide each drug for home-based treatment. BEAM: carmustine, Etopophos, cytarabine, melphalan; DA-EPOCH/EPOCH: rituximab, Etopophos, prednisolone, vincristine, cyclophosphamide, doxorubicin; DHAP: dexamethasone, high-dose arabine/cytarabine, cisplatin. HD: high dose; LD: low dose

The distribution of types of home-based treatments varies across the nine hospitals (Fig. 1). This tendency persists when separating into the different treatments provided as home-based treatment (Fig. 2). The UH provides six (UH3 and UH5) or five (UH4, UH1, and UH2) types of home-based treatments. One of the RHs provides the same types of treatment as three of the UHs (RH1). All hospitals provide immunoglobulin and antibiotics as home-based treatment. The least provided type of home-based treatment is hydration in continuation of chemotherapy.

**Fig. 3:**
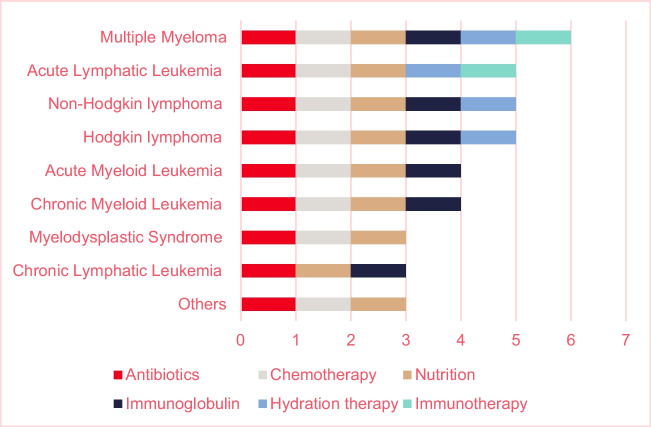
Types of home-based treatment across diagnosis

In total, 17 different treatments are administered in the patients’ homes across the nine hospitals, and the majority are implemented as standard practice (Table 2). Patients with MM have the most options for home-based treatments, followed by patients with lymphoma (HL and non-HL), and ALL (Fig. 3).

**Table 2:** Clinical characteristics of home-based treatments

Drug/treatment	Diagnosis	Year of introduction	Saved hospital visits/inpatient days per cycle	Estimated number of treatments in 2023
Chemotherapy				
HD Cytarabin	AML, ALL, non-HL, MDS, CMML	2015	> 5 inpatient days	115
Carfilzomib	MM, plasma cell leukemia	2017	3–4 visits	42
BEAM	Non-HL, HL	2017	1–2 inpatient days	21
DA-EPOCH/EPOCH	Non-HL	2019	5 inpatient days	23
LD Cytarabine	AML, MDS	2010	> 5 visits	45
Bortezomib	MM	2019	2–4 visits	120
Azacitidine	AML, MDS, CMML, T-cell lymphoma	2015	> 5 visits	247
Immunotherapy				
Daratumumab	MM	2021	1–4 visits	158
Elranatamab	MM	2024	3 visits	None
Blinatumomab	ALL	2015	> 5 visits	10
Hydration therapy				
HD Methotrexate	Non-HL	2019	3 to > 5 inpatient days	30
DHAP	Non-HL, HL	2022	2–4 inpatient days	11
Cyclophosphamide	ALL, non-HL, MM	2019	1 visit and > 5 inpatient days	100
Immunoglobulin				
Hizentra	AML, ALL, non-HL, MM, MDS, CMML, CLL	2007	1–5 visits	68
HyQvia	AML, ALL, non-HL, MM, MDS, CMML, CLL	2010	1–5 visits	333
Nutrition				
Parenteral nutrition	AML, ALL, non-HL, MM, MDS, CMML, CLL	N/A	N/A	21
Antibiotics				
Piperacillin/tazobactam	AML, ALL, non-HL, MM, MDS, CMML, CLL	2010	N/A	N/A

*AML* acute myeloid leukemia, *ALL* acute lymphatic leukemia, *CLL* chronic lymphatic leukemia, *CML* chronic myeloid leukemia, *MM* multiple myeloma, *non-HL* non-Hodgkin lymphoma, *HL* Hodgkin lymphoma, *MDS* myelodysplastic syndrome, *HD* high dose, *LD* low dose, *BEAM* carmustine–Etopophos–cytarabine–melphalan, *DA-EPOCH/EPOCH* retuximab–Etopophos–prednisolone–vincristine–cyclophosphamide–doxorubicin, *DHAP* dexamethasone, high dose, arabine/cytarabine, cisplatin, *N/A* not applicable

All hospitals provide formal educational programs for patients and their caregivers prior to the initiation of home-based treatment. However, no national consensus or guidelines exist regarding eligibility criteria for home-based treatment, including biochemical parameters as well as requirements for storage, dispensation, and administration. Consequently, substantial variation exists across hospitals.

### Chemotherapy

Seven different treatments of chemotherapy are provided as home-based treatment either intravenously (*n* = 4) or subcutaneously (*n* = 3) (Table 2). Most treatments are dispensed from the hospital or the hospital pharmacy, and for bortezomib and azacitidine, there is an option for patients to have them delivered at home as pre-filled syringes. Either the patient, a caregiver, or a primary care nurse can administer the subcutaneous treatments (bortezomib, azacitidine, and low-dose cytarabine). These treatments have specific requirements, including stability and storage temperature, which necessitate daily collection at the hospital or delivery during the treatment period. In contrast, the intravenous treatments (cytarabine, BEAM, and DA-EPOCH/EPOCH) are both dispensed and administered by nurses in the hospital´s hematology department. Following administration, the patients are sent home with the treatment connected to their central venous catheter (CVC) via a portable infusion pump (CADD pump). The pump is preprogrammed to deliver the treatment at specific time points, and patients return to the hospital either to have the infusion bag replaced or to be disconnected from the treatment. Exceptionally, for Carfilzomib, the patient or caregiver is responsible for connecting the treatment to the pre-installed CADD pump onto the patient’s peripheral venous catheters (PVC) at home. In general, the treatments are offered as home-based treatment from the first treatment cycle, except for Carfilzomib, which is not offered until the second cycle. Hospitals have different practices for administration time for intravenous treatments (< 30 to > 180 min) depending on diagnosis and protocols (data not shown). The number of administrations and consequently the number of reduced outpatient visits or inpatient days varies between treatments, see Table 2.

### Hydration therapy

Two hospitals provide hydration therapy in combination with three different types of chemotherapy: HD methotrexate (HD-MTX), DHAP (dexamethasone, HD arabine/cytarabine, and cisplatin), and cyclophosphamide (Fig. 2). Most hydration therapies are dispensed and administered at the hospital where, after the patient is sent home, the treatment is administrated at home on a CADD pump connected to a PVC, CVC, or peripherally inserted central catheter (PICC-line). The pump is preprogrammed to deliver the hydration, and patients return to the hospital either to have the infusion bag replaced or to be disconnected from the treatment. Hydration therapies are provided at home from the first treatment cycle and are all long-term infusions (> 180 min). The number of administrations varies from 3 to > 5 administrations per treatment cycle, with between 2 and > 5 reduced inpatient days (Table 2).

### Immunotherapy

Three types of immunotherapies (daratumumab, blinatumomab, and elranatamab) are provided as home-based treatment at five hospitals. Elranatamab is offered at one hospital on a trial basis for patients with MM. These immunotherapies are dispensed from the hospital or the hospital pharmacy. Daratumumab is administered as a subcutaneous injection. It can be delivered as a pre-filled syringe for each treatment, but at one hospital, the patient or caregiver is trained to pre-fill the syringe at home. The treatment can be administered by the patient, caregiver, or primary care nurses. There is variation between hospitals in relation to whether the treatment can be provided at home from the first, second, or third treatment cycle. Blinatumomab is infused intravenously (> 180 min) through a CVC or a PICC-line by using a CADD pump. The treatment is dispensed and administered by a nurse at the hospital, and the patient receives the treatment while being home. It can be provided from the first cycle of treatment with more than five administrations per treatment cycle, and therefore, more than five reduced hospital visits per treatment cycle. The infusion bag is not changed while the patient is at home; the pump stops automatically when the programmed infusion is completed, and trained healthcare professionals perform any bag changes exclusively at the hospital.

### Immunoglobulin

Immunoglobulin, as a home-based treatment, is delivered from the hospital department, the hospital pharmacy, or the local hospital, and is dispensed and administered by either the patient, their caregiver, or a primary care nurse. The treatment is administered subcutaneously using a Freedom pump (HyQvia, Hizentra) or CRONO S-PID/SoConnect (Hizentra). It varies between hospitals how early home-based treatment can be provided (first to third cycle of treatment), administration time (< 30 min up to 180 min), and number of reduced hospital visits (1 to > 5 visits).

### Nutrition

In most hospitals, treatment with parenteral nutrition is delivered as home-based treatment and can be dispensed and administered by the patient, their relatives, primary care nurses, or the hospital. The treatment is generally provided by the hospital or the hospital pharmacy, but in case of long-term treatment, other specialist hospital departments or primary care settings, including general practitioners, may be involved. The treatment is administered intravenously through a PVC, CVC, Midline, or PICC-line catheter.

### Antibiotics

Piperacillin/tazobactam is dispensed at the hospital or hospital pharmacy, after which the patient is sent home with the treatment connected to either a PVC, CVC, Midline, or PICC-line catheter. All hospitals use CADD pumps except UH1 and RH1, which use elastomer pumps. The treatment can be administered by the patient, their relatives, primary care nurses, or at the hospital. The latter requires the patient to visit the hospital to change the infusion bag with antibiotics. It varies between hospitals how early the antibiotic treatment can be initiated as home-based treatment from the first, second, or third dose.

### Patient criteria for receiving home-based treatment

Variation was observed in the criteria for receiving home-based treatment across both UHs and RHs. Most hospitals require patients to have cognitive and motoric resources to receive home-based treatment, especially if the treatment is administered subcutaneously. There is consensus that patients should be able to respond to potential side effects and complications. If an infusion pump is used, the criteria include being able to perform hygiene around the intravenous entry port, react to pump alarms, and correctly follow instructions through the telephone if problem-solving is needed. Moreover, the patients must be capable of measuring their temperature, drinking 2.5 L of fluid per day, taking oral medication as prescribed, and performing personal hygiene. Finally, the geographical distance to the hospital can be an exclusionary factor in some hospitals. For cytarabine, two hospitals have specific patient criteria, such as having a caregiver at home while receiving treatment, being less than 65 years old, and residing within a maximum distance of 50–100 km to the hospital. Conversely, one hospital did not have specific criteria for home-based treatment with rituximab, Etopophos, prednisolone, vincristine, cyclophosphamide, and doxorubicin (DA-EPOCH/EPOCH) and carmustine, Etopophos, cytarabine, and melphalan (BEAM).

According to the healthcare professionals, patients can refuse home-based treatment if they feel insecure about receiving treatment at home, are reluctant to administer treatment independently, reluctant to receive assistance from primary care nurses, reluctant to have their private home hospitalized, or due to geographical challenges. However, in two hospitals, treatments such as azacitidine and piperacillin/tazobactam are only offered as home-based treatment and therefore will be administered by primary care nurses if patients are not comfortable managing the treatment themselves.

## Discussion

### Discussion of findings

This nationwide explorative study is, to our knowledge, the first to assess the extent and practice of home-based treatment for patients with hematologic malignancies. Our results show that home-based treatment is widely implemented in the treatment of hematologic malignancies in Denmark. Several treatments are offered, with the majority being provided to patients with MM, while immunoglobulin and antibiotics are the most frequent types of treatment provided at home.

Our findings reflect the rapid evolution of home-based treatment within the field of hematology. Yet it shows that this advancement has been implemented differently across the country, increasing the risk of inequality in access to receiving treatment at home. Specifically, variations were identified in the eligibility criteria for patients receiving home-based treatment. Some hospitals have well-defined criteria, while others lack clearly defined criteria. To our knowledge, there are no national or international guidelines for home-based treatment within hematology, which may contribute to inconsistent practices and potentially suboptimal patient outcomes. In this context, the lack of clear and uniform criteria increases the likelihood that the healthcare professionals independently decide whether a patient is offered home-based treatment. Studies have shown that healthcare professionals often rely on their own assumptions when evaluating patients’ resources instead of actively involving the patients themselves [25, 26]. This could result in being less likely to recommend home-based treatment to individuals perceived––but not confirmed––as having limited resources [27]. Additionally, health narratives that emphasize patient proactivity and self-responsibility tend to favor those who are socially and physically advantaged, inadvertently marginalizing less privileged groups [28]. This emphasizes a need for tailored solutions, including caregiver training, financial assistance, and telehealth tools, to expand access to a broader population.

In this study, some hospitals require a caregiver at home while the patient receives the treatment. Although research on the impact of cancer-related home infusion therapies on caregivers is scant [29], a Danish study shows that home-based treatment with a portable chemotherapy pump for patients with AML is not of major concern to the caregivers [18]. The fact that the patient can stay at home is positive. Still, the practicalities at home and the responsibility regarding the pump and leukopenia demand the caregivers’ resources. Therefore, sufficient support and education are needed for both the patient and the caregiver [18]. Furthermore, the healthcare professionals at hospitals must identify patients’ and potential caregivers’ needs, instruct them to manage possible side effects during the treatment, and ensure communication that gives patients a sense of reassurance [30].

Our findings reveal substantial variations in home-based treatment practices across hospitals, underscoring the critical need for developing international guidelines tailored to hematological settings and enhancing disease management strategies, potentially improving the overall care trajectories for patients with hematological malignancies. Several countries have established policies supporting HaH programs, which may serve as models for developing such guidelines in Denmark. A recent paper on HaH care models suggests a validated taxonomy to ensure the safety and effectiveness of HaH [31]. This taxonomy may help identify which criteria should be prioritized when developing national or international guidelines for home-based treatment within hematology. Overall, international examples demonstrate that structured HaH policies can support safe, patient-centered care while reducing variation in clinical practice [32–35]. Such models could be adapted in Denmark to provide home-based treatment for patients with hematologic malignancies, including those receiving chemotherapy, ensuring equitable access and more standardized care. Moreover, the development of such a guideline could be based on a comprehensive review of the available data on home-based treatment. Currently, there are no systematic reviews available on home-based treatment interventions for hematological malignancies [10]. Specifically, this emphasizes the need for further investigation concerning home-based treatment, specific delivery models, and best practices for clinical and administrative management, including cost-effectiveness, potential, and challenges in cross-sector collaboration.

Home-based chemotherapy offers many benefits, including patient comfort, reduced hospital visits, and potentially lower healthcare costs [10, 11, 18, 19]. These benefits require close collaboration between many different healthcare professionals across sectors. Thus, successful implementation relies on strong interdisciplinary collaboration among healthcare professionals and intersectoral cooperation across various sectors, such as public health, community resources, and technology providers. Beyond the healthcare team, home-based treatment requires coordination across sectors to address logistical, technological, and social challenges. Barriers and facilitators to integrated cancer care between primary and secondary care have been identified [36]. To ensure efficient collaborative cancer care across sectors, a need for further training for primary care providers, clearly defined roles for healthcare professionals, effective communication and engagement between primary and secondary healthcare, and provision of guidelines is proposed [36–38]. However, further research is needed to better understand these barriers and facilitators to home-based treatments between primary and secondary care.

Effective registration practices are fundamental to the success of implementing home-based treatment [39, 40]. We identified inconsistencies in the registration of home-based treatment in the electronic health record (EHR) systems, which reduces the possibility of obtaining a valid overall view of the prevalence of home-based treatment. Moreover, the evidence emphasizes that practices for recording home-based treatments vary significantly between health sectors influenced by differences in resources and EHR systems [41, 42]. Lack of access to advanced EHR systems that support remote monitoring and data collection during home-based treatment can result in fragmented or delayed data entry, hindering real-time decision-making and compromising data interoperability and patient safety during transitions between care settings [41, 43]. Several studies have demonstrated that blockchain applications for health identity management, like MediLinker, improve interoperability by allowing patients to securely share their medical data with multiple providers through a web or mobile application [44–48]. Leveraging blockchain technology enables seamless interaction between different platforms, facilitating the use of identity data across various sectors [44, 45]. However, like other innovative technologies, blockchain faces challenges in achieving widespread adoption. Thus, comprehensive registration when receiving home-based treatment enables hospitals to analyze outcomes, track adherence to clinical guidelines, and identify areas for improvement [39, 40]. Continued development and scaling are necessary for this technology to reach its full potential and revolutionize modern healthcare practices. These practices are critical for ensuring patient safety, monitoring treatment efficacy, maintaining accurate records, and facilitating quality control and research on home-based treatment in patients with hematologic malignancies [39].

Despite the increasing evidence supporting home-based treatment for patients with hematologic malignancies, future studies investigating the feasibility, safety, registration practices, organization, and costs of home-based versus traditional hospital treatment are needed. Moreover, we lack evidence of the advantages and disadvantages for patients, relatives, and health professionals. This knowledge can potentially provide evidence-based recommendations for the development of clinical guidelines and future optimization of home-based care for patients with hematologic malignancies, including ensuring equal access to home-based treatment. Establishing robust evidence based on how home-based treatment can be most effectively organized for the benefit of patients, relatives, and healthcare professionals may pave the way for an international implementation of home-based care.

### Discussion of methods

The strength of the study is the nationwide and broad sample of included hematological departments in Denmark, potentially increasing the representativeness and generalizability of the findings. The results include treatments for various hematologic malignancies, which further strengthen the applicability of the findings. Finally, the national approach validates and supports the development of guidelines for the clinical implementation of home-based treatment for hematological malignancies. The data were collected using a questionnaire developed for this purpose, and it is a limitation that it was not possible to use a validated questionnaire. Conversely, it was an advantage that the author group collectively covered all regions in Denmark and could therefore assist the individual departments with the questionnaire, which is a strength explaining the high response rate. The total number of home-based treatments provided in 2023 is an estimate and should be interpreted with caution, as the documentation systems vary across the hospitals, and it is often not possible to document systematically and consistently whether the treatment was provided at the hospital or in the patient’s home. Finally, the generalizability to other countries may be affected by the degree of social, demographic, economic, and medical literacy heterogeneity. Despite these limitations, we believe that our study provides valuable insight into the implementation of home-based treatment across the Danish university and regional hospitals.

## Conclusion

In conclusion, this study highlights that home-based treatment for patients with hematologic malignancies is widely used in Denmark, revealing both the opportunities and challenges associated with its implementation. While the potential benefits of home-based treatment are significant, addressing the restrictions in access, standardizing practices, and enhancing support for patients and caregivers are essential steps toward optimizing and equalizing care delivery. The findings underscore the need for continued research, collaboration, and policy development to ensure that all patients have equitable access to high-quality home-based treatment options. By fostering a patient-centered approach and leveraging innovative technologies, we can pave the way for a more effective and compassionate healthcare system that meets the needs of patients with hematologic malignancies.

## Data Availability

No datasets were generated or analysed during the current study.

## Declarations

### Clinical trial registration number

Not relevant.
